# Longitudinal imaging correlates of stereotactic radiosurgery for refractory trigeminal neuralgia: A case report of rapid pain relief with 18-month follow-up

**DOI:** 10.1016/j.radcr.2025.06.100

**Published:** 2025-07-26

**Authors:** Moustafa A. Mansour, Mohamed Abdel-Fattah El-Salamoni, Hamdi Nabawi Mostafa

**Affiliations:** a Department of Neurosurgery, Nasser Institute for Research and Treatment, Cairo, Egypt; b Department of Human Physiology and Basic Medical Sciences, Faculty of Medicine, Al-Azhar University, Cairo, Egypt; c Department of Neurosurgery, Misr University for Science and Technology, Giza, Egypt

**Keywords:** Trigeminal neuralgia, Stereotactic radiosurgery, Facial pain

## Abstract

Stereotactic radiosurgery (SRS) is a well-established treatment for refractory trigeminal neuralgia (TGN), but longitudinal imaging data correlating radiological changes with clinical outcomes remain limited. We present a 79-year-old male with idiopathic TGN involving the right V2 trigeminal nerve distribution, refractory to pharmacotherapy and prior trigeminal ganglion balloon compression. Using an advanced plate-based SRS system, a precision dose of 90.0 Gy was delivered to the trigeminal nerve root entry zone (REZ). The patient reported complete pain relief within 3 days, sustained through 18-month follow-up, with mild sensory changes (paresthesia, numbness) considered an acceptable trade-off for pain freedom. Serial MRI follow-up at 4, 6, 12, and 18 months demonstrated dynamic post-SRS inflammatory changes at the REZ—initial enhancement peaking at 6–12 months followed by resolution at 18 months—providing rare radiological insights into treatment response. This case reinforces SRS as an effective option for refractory TGN, particularly in elderly patients, while highlighting the prognostic value of longitudinal imaging to bridge gaps in understanding post-procedural radiological evolution.

## Introduction

TGN, also known as tic douloureux, is a debilitating chronic pain disorder characterized by recurrent, paroxysmal episodes of severe, shock-like facial pain along the distribution of the trigeminal nerve. The pain is typically unilateral and most commonly affects the maxillary (V2) and mandibular (V3) divisions of the trigeminal nerve [1]. TGN is often triggered by innocuous stimuli such as light touch, chewing, or speaking, significantly impairing the patient's quality of life. The condition is more prevalent in individuals over the age of 50, with a slight female predominance [2].

The pathophysiology of TGN is multifactorial, with the most common cause being neurovascular compression of the trigeminal nerve REZ by adjacent blood vessels, leading to demyelination and aberrant neuronal signaling [3]. However, in cases where no vascular compression or structural lesion is identified, the condition is classified as idiopathic TGN. The pain in TGN is thought to arise from hyperexcitability of the trigeminal nerve, resulting in ectopic action potentials and central sensitization [4].

The management of TGN typically begins with pharmacological therapy, including anticonvulsants such as carbamazepine and gabapentin, which are effective in approximately 70%-80% of patients [5]. However, over time, many patients experience diminishing efficacy of medications or intolerable side effects, necessitating alternative interventions. For refractory cases, surgical options such as microvascular decompression (MVD), percutaneous rhizotomy, and SRS are considered. SRS, in particular, has emerged as a minimally invasive and effective treatment modality, especially for elderly patients or those who are not suitable candidates for more invasive procedures [6].

SRS is indicated for patients with medically refractory TGN, those who are poor surgical candidates, or those who prefer a non-invasive approach. The procedure involves delivering a highly focused dose of radiation to the trigeminal nerve root entry zone, targeting the site of presumed neurovascular compression or demyelination. The goal is to disrupt aberrant pain signaling while preserving neurological function. SRS is particularly advantageous in elderly patients, as it avoids the risks associated with general anesthesia and open surgery [7].

The advantages of SRS in treating TGN include its non-invasive nature, precision in targeting the trigeminal nerve, and low risk of major complications. Studies have demonstrated that SRS provides significant pain relief in approximately 70-90% of patients, with complete pain relief achieved in about 50%-60% of cases [6,8]. Additionally, SRS is associated with a lower risk of sensory deficits compared to other surgical interventions, making it an attractive option for patients seeking to avoid permanent numbness or other sensory complications [7]. The procedure is typically well-tolerated, with most adverse effects being mild and transient, such as temporary facial paresthesia or numbness.

While the clinical efficacy of SRS for TGN is well-documented [9], longitudinal imaging correlates of treatment response remain underexplored. Serial radiological follow-up can provide critical insights into post-SRS inflammatory changes, their temporal evolution, and potential association with clinical outcomes such as pain relief or sensory complications. This case report not only demonstrates the therapeutic benefits of SRS but also contributes unique longitudinal imaging data over 18 months—a feature seldom highlighted in existing literature—to better understand the anatomical and functional changes underlying successful treatment.

## Case presentation

A 79-year-old male presented with a 3-year history of severe, recurrent, and debilitating facial pain localized to the right side of his face, specifically involving the V2 (maxillary) division of the trigeminal nerve. The pain was characterized as sharp, shock-like, and paroxysmal, often triggered by light touch, chewing, or even speaking. The patient rated the pain as a 10/10 on the visual analog scale (VAS), indicating maximal severity. The episodes were frequent and significantly impacted his quality of life, making daily activities nearly impossible.

The patient had undergone extensive pharmacological management prior to seeking further intervention. This included trials of various analgesics and anticonvulsant medications, such as carbamazepine and gabapentin, which are commonly used to manage neuropathic pain. However, these treatments provided only partial and temporary relief, and the pain eventually became refractory to medical therapy. Approximately 2 years before the current presentation, the patient underwent trigeminal ganglion balloon compression which initially provided significant pain relief. However, the pain recurred after 2 years, prompting the need for a more definitive treatment approach.

A detailed neurological examination was performed, which was unremarkable aside from the facial pain. There were no focal neurological deficits, and the cranial nerve examination, including the trigeminal nerve motor and sensory functions, was normal. Imaging studies, including MRI, ruled out structural causes such as tumors or vascular compression, confirming the diagnosis of idiopathic trigeminal neuralgia.

Given the patient’s age, the refractory nature of his pain, and the failure of prior interventions, a multidisciplinary team decided to proceed with SRS as the next step in his management. SRS was chosen due to its minimally invasive nature, precision, and favorable outcomes in treating idiopathic trigeminal neuralgia, particularly in elderly patients who may not be ideal candidates for more invasive surgical procedures.

**Fig. 1:**
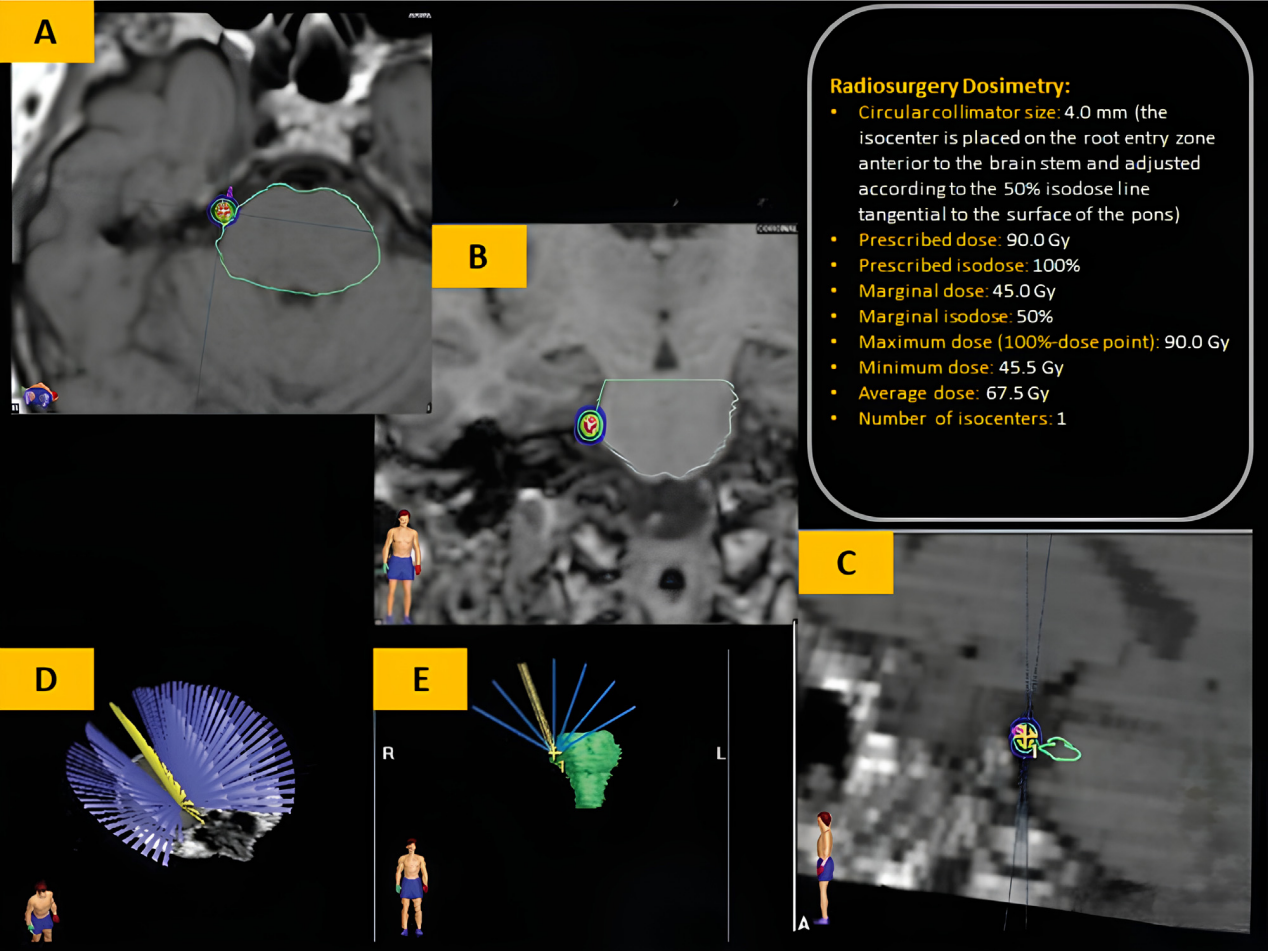
**SRS Treatment Plan.** Axial (A), coronal (B), and sagittal (C) T1-weighted images at the level of the trigeminal nerve. The SRS isocenter (*multicolored outline*) is placed on the trigeminal nerve root, 2-4 mm anterior to the point where the nerve emerges from the pons (*green outline*). The isocenter is adjusted according to the 50% isodose line, which is tangential to the surface of the pons (D and E).

The SRS treatment was meticulously planned and delivered using a plate-based system (Fig. 1). The target was the trigeminal nerve REZ, where the nerve emerges from the pons. A circular collimator with a diameter of 40 mm was used, and the isocenter was precisely placed at the root entry zone adjacent to the brainstem. The treatment delivered a precision dose of 90.0 Gy, with a marginal dose of 45.0 Gy, ensuring maximal therapeutic effect while minimizing radiation exposure to surrounding structures.

The patient’s response to SRS was remarkable. Within 3 days of the procedure, he reported complete resolution of his pain and was able to discontinue all pain medications. This pain-free state persisted throughout the follow-up period, with assessments at 4 months, 6 months, 12 months, and 18 months post-SRS confirming that the patient remained entirely free of pain and off medication.

However, the patient did experience some sensory changes in the treated area. At 6 months post-SRS, he reported a mild crawling sensation (paresthesia) in the V2 and V3 territories on the right side of his face. This sensation intensified slightly by 12 months but began to diminish by 18 months, at which point the patient also noted slight numbness in the same regions. These sensory changes were consistent with the expected effects of SRS on the trigeminal nerve and were deemed acceptable given the complete resolution of his debilitating pain.

**Fig. 2:**
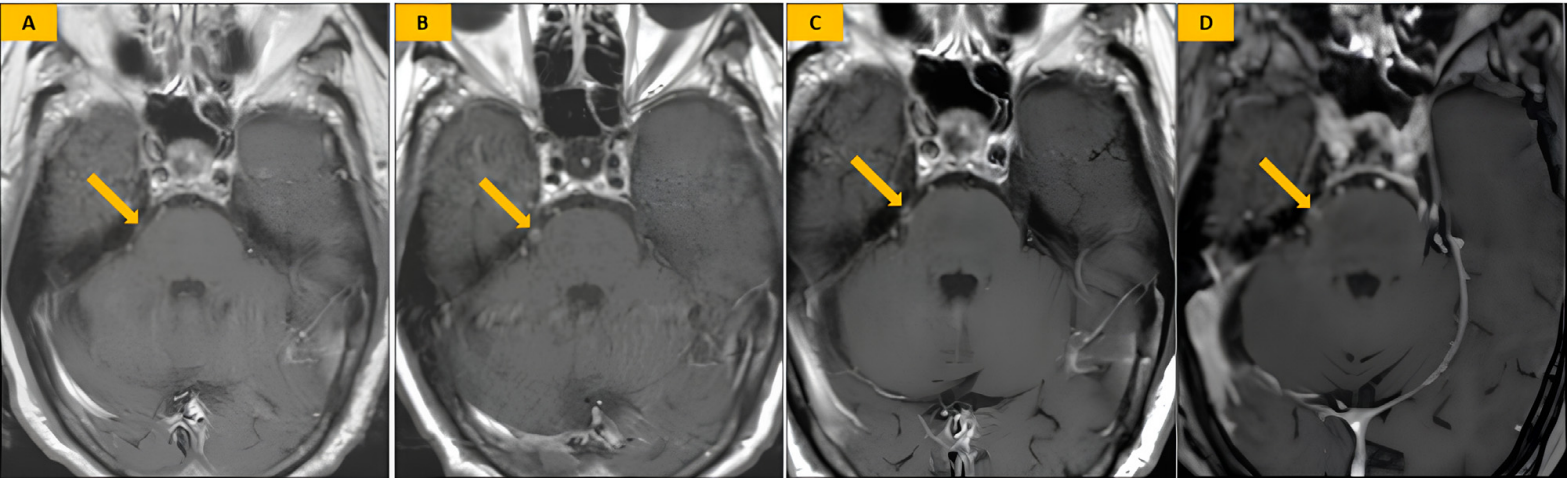
**Post-SRS Imaging.** (A) Axial T1-weighted contrast-enhanced image at 4 months post-SRS shows no significant imaging changes (*arrow*). (B) Axial T1-weighted contrast-enhanced image at 6 months post-SRS reveals enhancement of the right trigeminal root entry zone (*arrow*). (C) Axial T1-weighted contrast-enhanced image at 12 months post-SRS shows stationary enhancement of the right trigeminal root entry zone (*arrow*). (D) Axial T1-weighted contrast-enhanced image at 18 months post-SRS demonstrates decreased enhancement of the right trigeminal root entry zone along with an emerging focus of encephalomalacia (*arrow*), consistent with expected post-treatment changes. The apparent left-sided enhancement likely represents volume averaging of adjacent petrosal venous structures rather than true pathology, as the patient remained asymptomatic on the contralateral side throughout follow-up.

Serial MRI scans were performed to monitor the radiological changes following SRS. At 4 months post-SRS, no significant imaging changes were observed (Fig. 2**A**). By 6 months, there was evidence of enhancement at the right trigeminal nerve entry zone into the pons on T1 gadolinium-enhanced imaging, likely reflecting post-radiation inflammatory changes (Fig. 2**B**). This enhancement remained stable at 12 months (Fig. 2**C**) but showed a noticeable decrease by 18 months, suggesting a resolution of the inflammatory response (Fig. 2**D**).

The patient experienced mild hyperesthesia (increased sensitivity to touch) in the V2 and V3 territories at 1-month post-SRS, but this complication resolved spontaneously over time. No other significant adverse effects were reported, and the patient tolerated the treatment well.

The patient’s outcome was highly favorable, with complete pain relief and no need for further pharmacological intervention. The mild sensory changes (paresthesia and numbness) were considered a reasonable trade-off for the significant improvement in his quality of life. Continued clinical and radiological follow-up was recommended to monitor for any late complications or recurrence of symptoms.

## Discussion

This case report highlights the successful management of idiopathic TGN in a 79-year-old male using SRS, with a unique emphasis on longitudinal imaging correlates over an 18-month follow-up period. The patient achieved complete pain relief within 3 days of the procedure, sustained through serial evaluations, reinforcing SRS as an effective treatment for refractory TGN, particularly in elderly patients.

The temporal pattern of radiological changes observed in this case provides novel insights into post-SRS inflammatory responses. Serial MRI revealed enhancement at the trigeminal nerve REZ peaking at 6–12 months, followed by resolution at 18 months. This dynamic evolution paralleled the patient’s clinical course—rapid pain relief followed by mild, stable sensory changes—suggesting that inflammatory changes may serve as an imaging biomarker for treatment response. While prior studies have established the efficacy of SRS for TGN [6,8], few have documented such detailed longitudinal imaging correlations, making this a valuable contribution to the literature.

The patient’s prior failure to respond to pharmacotherapy and balloon compression underscores the challenges of managing refractory TGN. SRS offers a compelling alternative, especially for elderly patients who may not tolerate invasive procedures. The observed sensory changes (paresthesia and numbness) were mild and deemed an acceptable trade-off for pain relief, consistent with prior reports [6]. Notably, these sensory effects emerged after the peak of radiological enhancement, implying a potential predictive relationship between imaging findings and late sequelae.

Our imaging findings align with the proposed mechanisms of SRS—focused radiation-induced modulation of hyperexcitable trigeminal nerve fibers at the REZ. The transient enhancement likely reflects localized inflammation and demyelination, which subsided as the nerve stabilized. This supports the hypothesis that SRS disrupts aberrant pain signaling while preserving structural integrity, a balance critical for optimizing outcomes.

While this case demonstrates excellent results, it is important to acknowledge limitations. The single-patient design precludes broad generalizations, and the imaging findings, though compelling, require validation in larger cohorts with standardized protocols. Nevertheless, the consistency between radiological and clinical progression strengthens the argument for incorporating routine imaging follow-up in SRS-treated TGN patients.

## Conclusion

This case illustrates the efficacy of SRS in achieving rapid and durable pain relief for refractory idiopathic TGN, particularly in elderly patients. Beyond clinical outcomes, the longitudinal imaging data—showing reversible inflammatory changes at the REZ—provides a rare window into the biological effects of SRS, enhancing our understanding of treatment mechanisms. While mild sensory changes remain a trade-off, their manageable nature and the non-invasive profile of SRS solidify its role as a first-line intervention for medically refractory cases. Future studies should prioritize systematic imaging follow-up to validate these observations and explore whether early radiological changes predict long-term outcomes. Such efforts could refine patient selection, improve counseling, and optimize procedural parameters. Until then, this case underscores the dual value of SRS—as both a therapeutic tool and a subject of scientific inquiry into neuropathic pain modulation.

## Contribution

M.M. was responsible for the conception of the work, data collection, drafting the article, critical revisions, and obtaining approval of the final version of the manuscript. M.E. and H.M. contributed by drafting the article, and critical revisions. All authors read the final manuscript and were involved in direct patient care.

## Patient consent

The authors declare that they have obtained consent from the patient.
